# Predictors of Fear of Childbirth in Nulliparous Women: Childhood Traumas and Attachment Patterns

**DOI:** 10.5964/ejop.18653

**Published:** 2026-05-29

**Authors:** Gökçe Kavak Sinanoğlu, Sezin Ateş

**Affiliations:** 1 Department of Psychiatry, Alanya Alaaddin Keykubat University Alanya Education and Research Hospital, Antalya, Türkiye; 2 Department of Obstetrics and Gynecology, Alanya Alaaddin Keykubat University Alanya Education and Research Hospital, Antalya, Türkiye; Birmingham City University, Birmingham, United Kingdom

**Keywords:** fear of childbirth, childhood trauma, attachment styles, nulliparous women

## Abstract

This study aimed to investigate the predictors of fear of childbirth (FoC) in nulliparous women by focusing on the effects of childhood trauma and attachment styles. Identifying psychological factors contributing to FoC is important for improving early intervention strategies and maternal well-being. The study included 496 nulliparous women who had never experienced pregnancy or childbirth. Participants completed the Women’s Childbirth Fear–Prior to Pregnancy Scale (WCF-PPS), the Childhood Trauma Questionnaire–Short Form (CTQ-SF), and the Experiences in Close Relationships–Relationship Structures Questionnaire (ECR-RS). Pearson correlation and multiple linear regression analyses were conducted to examine the relationships between FoC, childhood trauma, and attachment styles. Fear of childbirth showed a significant positive correlation with anxious attachment (*r* = .257, *p* < .001). Weak but statistically significant associations were also found with emotional abuse (*r* = .094, *p* = .039) and total childhood trauma (*r* = .090, *p* = .049). However, in the regression analysis, only anxious attachment significantly predicted FoC (β = .172, *p* < .001), while childhood trauma and avoidant attachment did not emerge as significant predictors. Anxious attachment was identified as the most prominent psychological predictor of FoC in nulliparous women. These results suggest that addressing attachment-related anxiety and early traumatic experiences in pre-pregnancy or prenatal psychological evaluations may reduce fear of childbirth and support maternal mental health.

Pregnancy and childbirth represent significant transitions in a woman’s life, often accompanied by emotional and physical challenges. Experiencing anxiety regarding these consequences is quite common (Hofberg & Ward, 2003). Fear of childbirth, which may manifest at both clinical and subclinical levels, can be observed among women regardless of their parity (O’Connell et al., 2017). The prevalence of fear of childbirth varies considerably across studies, largely due to differences in definition and measurement methods, as well as cross-cultural factors. In Scandinavian countries, approximately 20% of women report general fear of childbirth, with 5–10% classified as severe. Across Europe, prevalence ranges between 1.9% and 14%, whereas in Australia it may reach 30% (Nilsson et al., 2018).

Fear of childbirth is a multifaceted phenomenon shaped by the interaction of biological, psychological, and social factors. Biological contributors include labor pain, the risk of operative interventions, and concerns about maternal or infant harm (Dencker et al., 2019). Psychological risk factors include anxiety, depression, low self-esteem, a history of traumatic births, or sexual abuse (Kananikandeh et al., 2022). Furthermore, lack of social support, low socioeconomic status, negative birth narratives, and mistrust in healthcare professionals may exacerbate this fear (Puşuroğlu, 2021). In nulliparous women, uncertainty about pain, loss of control during labor, and fears of harming the baby are particularly pronounced, often contributing to requests for cesarean delivery (Nieminen et al., 2009). For instance, a Norwegian study reported that 14–22% of elective cesareans occurred at maternal request (Størksen et al., 2015). Beyond influencing delivery preferences, childbirth fear is also associated with impaired maternal mental health, infant development, and postpartum adaptation (Klabbers et al., 2016). Thus, identifying its underlying mechanisms is of both clinical and public health relevance.

Among potential determinants, childhood trauma has received considerable attention. It is commonly categorized into five domains: emotional abuse, physical abuse, sexual abuse, emotional neglect, and physical neglect (Burgermeister, 2007). Research suggests that experiences of emotional and physical abuse increase the risk of fear of childbirth, whereas findings on sexual abuse remain inconsistent, and the role of neglect is still underexplored. Notably, fear of childbirth has been linked to diminished family well-being and increased rates of cesarean sections (Porthan et al., 2023). Therefore, investigating the contribution of childhood trauma may help clarify the origins of childbirth fear and inform intervention strategies.

Another relevant framework is attachment theory. According to Bowlby (1988), early relational experiences shape attachment styles, which in turn influence emotional regulation and coping throughout life. Stressful and emotionally charged periods such as pregnancy and childbirth can activate these attachment patterns, shaping women’s reactions (Reisz et al., 2019). Insecure attachment styles, particularly anxious and avoidant orientations, have been associated with heightened anxiety and childbirth-related fears (Yıldırım et al., 2025). A more nuanced understanding of the interplay between childhood trauma and attachment patterns may therefore shed light on individual differences in fear of childbirth.

The present study aims to examine the effects of childhood trauma and attachment patterns on fear of childbirth in nulliparous women. It was hypothesized that higher levels of childhood trauma and insecure attachment styles would predict greater fear of childbirth.

## Method

### Research Model

In this cross-sectional, observational study, the independent variables were childhood trauma experiences (assessed with the Childhood Trauma Questionnaire–Short Form, CTQ-SF) and attachment patterns (assessed with the Experiences in Close Relationships–Relationship Structures Questionnaire, ECR-RS). The dependent variable, fear of childbirth, was measured using the Women’s Childbirth Fear–Prior to Pregnancy Scale (WCF-PPS).

### Participants

The study was conducted with a total of 496 nulliparous women who presented to the Department of Obstetrics and Gynecology at Alanya Education and Research Hospital between January 2024 and January 2025. Participants were recruited on a voluntary basis. During their clinic visits, women were provided with study forms and completed them individually in a face-to-face procedure. Inclusion criteria were: never having given birth, no history of pregnancy, no history of abortion for any reason, willingness to participate in the study, and being literate. Exclusion criteria included: having previously given birth, having a history of miscarriage or abortion, and illiteracy.

### Measurement

#### Demographic Information

Sociodemographic data, including age, marital status, education level, employment status, and place of residence, were collected. These variables were considered relevant for understanding psychological responses during pregnancy and could influence fear of childbirth, thus enhancing the interpretability and replicability of the study.

#### Women’s Childbirth Fear–Prior to Pregnancy Scale (WCF-PPS)

Stoll et al. (2016) created the Childbirth Fear–Prior to Pregnancy Scale to gauge young men’s and women’s fear of childbirth before becoming pregnant. In 2018, Uçar and Taşhan translated the scale into Turkish. The scale consists of ten items, scored on a six-point Likert scale ranging from 1 to 6. Total scores range from 10 to 60, with higher scores indicating higher levels of fear. The Cronbach’s alpha value in Stoll et al.’s (2016) study was .86. Similar validity and reliability studies were conducted independently for the male and female versions of the scale by Uçar and Taşhan (2018). In this study, the Cronbach's alpha value of the scale was determined as .86.

#### Childhood Trauma Questionnaire–Short Form (CTQ-SF)

The CTQ-SF is a 28-item short form of the original 70-item scale (Bernstein et al., 2003). It has a Turkish validity and reliability study and consists of 25 questions across five subscales measuring childhood emotional, physical, and sexual abuse, as well as physical and emotional neglect, along with three items assessing denial. Total and subscale scores are calculated. Cut-off points suggested by Şar et al. (2012) were applied: 5 for sexual and physical abuse, 7 for physical neglect and emotional abuse, and 12 for emotional neglect and these cut-off scores were used in the study.

#### Experiences in Close Relationships–Relationship Structures Questionnaire (ECR-RS)

The ECR-RS is a multidimensional scale developed to evaluate individuals’ attachment relationships with various people (e.g., mother, father, romantic partner, close friend). The scale measures attachment anxiety and attachment avoidance separately for each relationship type. It consists of seven items: three items assess attachment anxiety, and four items assess attachment avoidance, using a Likert-type format (Fraley et al., 2011). The Turkish version was validated and proven reliable in 2021 (Deveci Şirin & Şen Doğan, 2021).

### Data Analysis

Data obtained in the study were analyzed using IBM SPSS Statistics Version 21.0. Descriptive statistics, including mean, standard deviation, minimum, and maximum values, were calculated for each variable. The Kolmogorov–Smirnov test was used to assess the normality of data distribution. Pearson correlation analysis was conducted to examine relationships between the independent variables and fear of childbirth. To test the predictive relationships between childhood trauma (CTQ-SF) and attachment dimensions (ECR-RS anxiety and avoidance) with fear of childbirth (WCF-PPS), multiple linear regression analysis was performed. For all analyses, a significance level of *p* < .05 was considered statistically significant.

### Transparency and Openness

Data, SPSS syntax for data preparation and analysis, codebook, original item wordings with English translations, and all materials used in the study are available at OSF in a folder titled “Predictors of Fear of Childbirth in Nulliparous Women” (Kavak Sinanoğlu, 2025). Data were analyzed using SPSS version 21 for this report. This study's design and its analyses were not preregistered.

## Results

The sample consists of 496 individuals, with a mean age of 25.17 years (*SD* = 7.08), ranging from 18 to 42 years. Regarding educational status, the majority of participants have attained at least a high school diploma, with 61.7% having completed university, 20.8% high school, 6.7% secondary school, and 5.8% primary school. A smaller proportion of participants, 5%, have completed postgraduate education. The sociodemographic characteristics of the participants are presented in Table 1.

**Table 1:** Sociodemographic Characteristics of Participants

Variable	*n*	%	*M* (SID)	Min–Max
Age	496	100	25.17 (7.08)	18–42
Education status				
Primary school	29	5.8		
Secondary school	33	6.7		
High school	103	20.8		
University	306	61.7		
Postgraduate	25	5.0		
Marital status				
Married	159	32.1		
Single	337	67.9		
Place of residence				
Urban	29	5.8		
Semi-urban	457	92.1		
Rural	10	2.0		

*Note*. *N* = number, SID = Standard Deviation.

Pearson correlation analyses were conducted to examine the relationships between childbirth fear (WCF-PPS) and various psychosocial variables including childhood trauma and attachment styles. The results revealed a significant positive correlation between WCF-PPS and anxious attachment (*r* = .257, *p* < .001), suggesting that individuals with higher levels of anxious attachment tend to report greater fear of childbirth.

In addition, childhood trauma total score (*r* = .090, *p* = .049) and emotional abuse (*r* = .094, *p* = .039) were also weakly but significantly correlated with childbirth fear. Other trauma subtypes, such as physical and sexual abuse, emotional and physical neglect, and avoidant attachment, did not show statistically significant associations with WCF-PPS (all *p* > .05). The Pearson correlations between WCF-PPS and CTQ-SF, ECR-RS are presented in Table 2.

**Table 2:** Pearson Correlations Between WCF-PPS and CTQ-SF, ECR-RS

Variable	*r*	*p*
CTQ-SF total	.090	.049 *
Emotional abuse	.094	.039 *
Physical abuse	.067	.140
Sexual abuse	.077	.091
Emotional neglect	.070	.124
Physical neglect	.050	.264
ECR-RS avoidant	.004	.931
ECR-RS anxious	.257	< .001 **

*Note.* WCF-PPS = Women’s Childbirth Fear – Prior to Pregnancy Scale; CTQ-SF = Childhood Trauma Questionnaire–Short Form; ECR-RS = Experiences in Close Relationships–Revised–Short Form.

*p < .05. **p < .001.

These findings indicate that anxious attachment and specific dimensions of childhood trauma, particularly emotional abuse, may play a modest role in influencing fear of childbirth among nulliparous women.

A multiple linear regression analysis was conducted to examine the predictive effects of childhood trauma (CTQ-SF total and subscales), attachment styles (ECR-RS_avoidant and ECR-RS_anxious), and age on birth fear (WCF-PPS). The regression model was found to be significant (*F* (9, 435) = 2.977, *p* = .002), and the model's variables explained approximately 5.8% of the variance in WCF-PPS scores (*R*^2^ = .058, adjusted *R*^2^ = .039).

Upon examining the regression coefficients, it was found that only the anxious attachment style (ECR-RS_anxious) significantly predicted birth fear (β = .172, *t* = 3.652, *p* < .001). This result indicates that as an individual’s attachment anxiety increases, their level of birth fear also significantly rises. On the other hand, the total score and subscales of childhood trauma (emotional abuse, physical abuse, sexual abuse, emotional neglect, physical neglect), avoidant attachment style (ECR-RS_avoidant), and age did not contribute significantly to the model (*p* > .05). Results of Multiple Linear Regression Analysis Predicting Birth Fear (WCF-PPS) are presented in Table 3.

**Table 3:** Regression coefficients for Predictors of Childbirth Fear (WCF-PPS)

Predictor	*B*	SE B	β	*t*	*p*
CTQ-SF total	0.197	0.518	0.108	0.380	.704
Emotional abuse	0.046	0.645	0.007	0.071	.943
Physical abuse	0.434	0.620	0.065	0.700	.484
Sexual abuse	–0.437	0.595	–0.092	–0.735	.463
Emotional neglect	0.249	0.663	0.034	0.375	.708
Physical neglect	–0.626	0.718	–0.109	–0.872	.383
Avoidant attachment	–0.049	0.074	–0.032	–0.667	.505
Anxious attachment	0.407	0.111	0.172	3.652	< .001 **
Age	–0.073	0.073	–0.047	–1.003	.316

*Note.* Dependent variable = WCF-PPS (Women’s Childbirth Fear – Prior to Pregnancy Scale).

*p < .05. **p < .001.

## Discussion

This study aimed to quantitatively examine the influence of individuals’ early traumatic experiences and attachment dynamics in close relationships on their levels of fear related to childbirth. Our findings revealed that anxious attachment was the strongest predictor of childbirth fear, whereas childhood trauma, particularly emotional abuse, exhibited a modest but significant association. These results underscore the importance of psychosocial factors in understanding fear of childbirth.

Consistent with our findings, a Finnish birth cohort study including 2,556 women reported that emotional abuse and emotional neglect significantly increased the risk of childbirth fear, whereas physical abuse, physical neglect, and sexual abuse were not statistically significant predictors (Porthan et al., 2023). Similarly, a study conducted in Norway with 2,365 pregnant women revealed that 18% of those with a history of childhood abuse experienced severe fear of childbirth, compared to 10% among those without such a history. This association was particularly evident among primiparous women (adjusted OR: 2.00; 95% CI: 1.30–3.08) (Lukasse et al., 2010). These findings support our results, suggesting that emotional abuse, in particular, may contribute to increased childbirth fear.

From the perspective of Bowlby’s attachment theory, attachment patterns appear to play a pivotal role in childbirth-related anxiety. In a study by Reisz et al. (2019), it was found that mothers with secure attachment styles reported higher levels of perceived support during childbirth and greater satisfaction with their birth experiences. The theoretical constructs of a “secure base” and a “safe haven” were emphasized as protective factors during the childbirth process.

Conversely, women with avoidant attachment styles have been reported to be at greater risk for experiencing postnatal trauma symptoms. In a recent study by Yıldırım et al. (2025), pregnant women with avoidant attachment styles were shown to have higher levels of childbirth fear compared to those with secure attachment styles. Furthermore, elevated anxiety levels and previous negative birth experiences were also cited as factors contributing to fear of childbirth. In our current study, while no statistically significant relationship was found between avoidant attachment and fear of childbirth, a significant association was observed specifically with anxious attachment. Bowlby defined anxious attachment as a pattern characterized by an intense need for closeness and a fear of abandonment, which develops as a result of inconsistent responses from caregivers (Bowlby, 1988). In adulthood, individuals with this attachment style tend to display excessive reassurance seeking, emotional fluctuations, and heightened separation anxiety in their relationships (Mikulincer & Shaver, 2016). These characteristics may increase anxiety and the need for support during childbirth — a process marked by uncertainty and loss of control — and thereby contribute to greater fear of childbirth. Golmakani et al. (2020) also reported that fear can increase attachment anxiety. This situation can create a cycle that feeds anxiety during the birth process.

### Limitations

This study has several limitations. Its cross-sectional design limits causal interpretations, as temporal relationships cannot be established. However, this design was chosen to provide an initial understanding of predictors of fear of childbirth in nulliparous women within a feasible timeframe. The use of self-report measures may have introduced response biases; nonetheless, validated instruments were employed to minimize this risk. The sample, drawn from a single center and limited to nulliparous women, restricts generalizability. Focusing on nulliparous women was intentional, as this group typically experiences fear of childbirth differently compared to multiparous women, making them a unique population of interest. Recruiting from a single center ensured homogeneity in procedures but reduced sample diversity. Additionally, some potentially influential factors such as current mental health status and social support were not assessed, partly to reduce participant burden and keep the questionnaire length manageable. Despite these limitations, the use of validated instruments and face-to-face data collection strengthened the reliability of the results.

### Conclusion

In conclusion, this research demonstrates that fear of childbirth is not solely related to biological or experiential factors but is also closely linked to early relational experiences and attachment patterns. Accordingly, considering women’s attachment styles and past traumatic experiences during prenatal care may contribute to the development of a more holistic and psychologically supportive care model. In particular, identifying the psychosocial support needs of individuals with anxious attachment styles may serve as a critical step toward reducing fears associated with childbirth.

## Supplementary Materials

**Table:** 

Type of supplementary material	Availability/Access
Data	
SPSS file	Kavak Sinanoğlu, 2025
Code	
SPSS syntax - Fear of childbirth	Kavak Sinanoğlu, 2025
SPSS code	Kavak Sinanoğlu, 2025
Material	
Original Turkish language study materials	Kavak Sinanoğlu, 2025
Table items - Original language and English	Kavak Sinanoğlu, 2025
Informed Consent Form (ICF)	Kavak Sinanoğlu, 2025
Study/Analysis preregistration	
Study was not preregistered	—
Other	
Codebook - Fear of childbirth	Kavak Sinanoğlu, 2025

## Data Availability

Data can be accessed through the Open Science Framework (Kavak Sinanoğlu, 2025).

## References

[r1] Bowlby, J. (1988). Developmental psychiatry comes of age. The American Journal of Psychiatry, 145(1), 1–10. 10.1176/ajp.145.1.1 3276225

[r2] Bernstein, D. P., Stein, J. A., Newcomb, M. D., Walker, E., Pogge, D., Ahluvalia, T., Stokes, J., Handelsman, L., Medrano, M., Desmond, D., & Zule, W. (2003). Development and validation of a brief screening version of the Childhood Trauma Questionnaire. Child Abuse & Neglect, 27(2), 169–190. 10.1016/S0145-2134(02)00541-0 12615092

[r3] Burgermeister, D. (2007). Childhood adversity: A review of measurement instruments. Journal of Nursing Measurement, 15(3), 163–176. 10.1891/106137407783095766 18232616

[r4] Deveci Şirin, H., & Şen Doğan, R. (2021). Psychometric properties of the Turkish version of the Experiences in Close Relationships–Relationship Structures Questionnaire (ECR-RS). SAGE Open, 11(1). 10.1177/21582440211006056

[r5] Dencker, A., Nilsson, C., Begley, C., Jangsten, E., Mollberg, M., Patel, H., Wigert, H., Hessman, E., Sjöblom, H., & Sparud-Lundin, C. (2019). Causes and outcomes in studies of fear of childbirth: A systematic review. Women and Birth : Journal of the Australian College of Midwives, 32(2), 99–111. 10.1016/j.wombi.2018.07.004 30115515

[r6] Fraley, R. C., Heffernan, M. E., Vicary, A. M., & Brumbaugh, C. C. (2011). The Experiences in Close Relationships–Relationship Structures Questionnaire: A method for assessing attachment orientations across relationships. Psychological Assessment, 23(3), 615–625. 10.1037/a0022898 21443364

[r7] Golmakani, N., Gholami, M., Shaghaghi, F., Safinejad, H., Kamali, Z., & Mohebbi-Dehnavi, Z. (2020). Relationship between fear of childbirth and the sense of cohesion with the attachment of pregnant mothers to the fetus. Journal of Education and Health Promotion, 9(1), 261. 10.4103/jehp.jehp_46_20 33282966 PMC7709774

[r8] Hofberg, K., & Ward, M. R. (2003). Fear of pregnancy and childbirth. Postgraduate Medical Journal, 79(935), 505–510. 10.1136/pmj.79.935.505 13679545 PMC1742837

[r9] Kananikandeh, S., Shokravi, F. A., Mirghafourvand, M., & Jahanfar, S. (2022). Factors of the childbirth fear among nulliparous women in Iran. BMC Pregnancy and Childbirth, 22(1), 547. 10.1186/s12884-022-04870-1 35794544 PMC9260972

[r10] Kavak Sinanoğlu, G. (2025). Predictors of fear of childbirth in nulliparous women: Childhood traumas and attachment patterns [OSF project page containing codebook, study dataset, SPSS syntax, and study materials]. OSF. https://osf.io/64jm8/overview

[r11] Klabbers, G. A., van Bakel, H. J. A., van den Heuvel, M. M. A., & Vingerhoets, A. J. J. M. (2016). Severe fear of childbirth: Its features, assessment, prevalence, determinants, consequences and possible treatments. Psihologijske Teme, 25(1), 107–127. https://hrcak.srce.hr/156335

[r12] Lukasse, M., Vangen, S., Øian, P., Kumle, M., Ryding, E. L., & Schei, B. (2010). Childhood abuse and fear of childbirth: A population-based study. Birth (Berkeley, Calif.), 37(4), 267–274. 10.1111/j.1523-536X.2010.00420.x 21083717

[r13] Mikulincer, M., & Shaver, P. R. (2016). *Attachment in adulthood: Structure, dynamics, and change* (2^nd^ ed.). Guilford Press.

[r14] Nieminen, K., Stephansson, O., & Ryding, E. L. (2009). Women’s fear of childbirth and preference for cesarean section: A cross-sectional study at various stages of pregnancy in Sweden. Acta Obstetricia et Gynecologica Scandinavica, 88(7), 807–813. 10.1080/00016340902998436 19488882

[r15] Nilsson, C., Hessman, E., Sjöblom, H., Dencker, A., Jangsten, E., Mollberg, M., Patel, H., Sparud-Lundin, C., Wigert, H., & Begley, C. (2018). Definitions, measurements and prevalence of fear of childbirth: A systematic review. BMC Pregnancy and Childbirth, 18, 28. 10.1186/s12884-018-1659-7 29329526 PMC5766978

[r16] O’Connell, M. A., Leahy-Warren, P., Khashan, A. S., Kenny, L. C., & O’Neill, S. M. (2017). Worldwide prevalence of tocophobia in pregnant women: Systematic review and meta-analysis. Acta Obstetricia et Gynecologica Scandinavica, 96(8), 907–920. 10.1111/aogs.13138 28369672

[r17] Porthan, E., Lindberg, M., Härkönen, J., Scheinin, N. M., Karlsson, L., & Karlsson, H. (2023). Childhood trauma and fear of childbirth: Findings from a birth cohort study. Archives of Women’s Mental Health, 26(4), 523–529. 10.1007/s00737-023-01328-x 37243781 PMC10333423

[r18] Puşuroğlu, M. (2021). Tokofobi [Tokophobia]. *Aksaray Üniversitesi Tıp Bilimleri Dergisi, 2*(3), 34–38. https://dergipark.org.tr/tr/pub/asujms/issue/67709/885556

[r19] Reisz, S., Brennan, J., Jacobvitz, D., & George, C. (2019). Adult attachment and birth experience: Importance of a secure base and safe haven during childbirth. Journal of Reproductive and Infant Psychology, 37(1), 26–43. 10.1080/02646838.2018.1509303 30269511 PMC6343367

[r20] Şar, V., Öztürk, E., & İkikardeş, E. (2012). Validity and reliability of the Turkish version of Childhood Trauma Questionnaire. Turkish Journal of Psychiatry, 32(4), 1054–1063. 10.5336/medsci.2011-26947

[r21] Stoll, K., Hauck, Y., Downe, S., Edmonds, J., Gross, M. M., Malott, A., McNiven, P., Swift, E., Thomson, G., & Hall, W. A. (2016). Cross-cultural development and psychometric evaluation of a measure to assess fear of childbirth prior to pregnancy. Sexual & Reproductive Healthcare : Official Journal of the Swedish Association of Midwives, 8(1), 49–54. 10.1016/j.srhc.2016.02.004 27179378

[r22] Størksen, H. T., Garthus-Niegel, S., Adams, S. S., Vangen, S., & Eberhard-Gran, M. (2015). Fear of childbirth and elective caesarean section: A population-based study. BMC Pregnancy and Childbirth, 15, 221. 10.1186/s12884-015-0655-4 26382746 PMC4573308

[r23] Uçar, T., & Taşhan, S. (2018). The Turkish version of the Childbirth Fear–Prior to Pregnancy Scale: The validity and reliability study in men and women. Anadolu Üniversitesi Sağlık Bilimleri Dergisi*, *9(3), 289–296. https://dergipark.org.tr/en/download/article-file/1701775

[r24] Yıldırım, Y. E., Çetinay Aydın, P., İnanç Ünlü, A., Karaca, İ., & Ekin, M. (2025). Comparison of state-trait anxiety and fear of childbirth according to attachment styles of pregnant women. The Journal of Perinatal & Neonatal Nursing, 39(4), 312–321. 10.1097/JPN.0000000000000844 39325946

